# Enhancing the Nutritional Properties of Bread by Incorporating Mushroom Bioactive Compounds: The Manipulation of the Pre-Dictive Glycaemic Response and the Phenolic Properties

**DOI:** 10.3390/foods10040731

**Published:** 2021-03-30

**Authors:** Xikun Lu, Margaret A. Brennan, Wenqiang Guan, Jie Zhang, Li Yuan, Charles S. Brennan

**Affiliations:** 1 Tianjin Key Laboratory of Food Biotechnology, College of Biotechnology and Food Sciences, Tianjin University of Commerce, Tianjin 300314, China; xikunlu@tjcu.edu.cn (X.L.); guanwenqiang@tjcu.edu.cn (W.G.); 2 Department of Wine, Food and Molecular Biosciences, Lincoln University, P.O. Box 84, Lincoln, Christchurch 7647, New Zealand; Margaret.Brennan@lincoln.ac.nz; 3 Institute of Food Science and Technology, Chinese Academy of Agricultural Sciences/Key Laboratory of Agro-Products Processing, Ministry of Agriculture, Beijing 100193, China; zhangjie@caas.cn (J.Z.); yuanli233@outlook.com (L.Y.); 4 School of Science, RMIT, Melbourne, VIC 3000, Australia

**Keywords:** mushroom bread, starch digestibility, antioxidant ability, microstructure

## Abstract

Wheat bread supplemented with mushroom powder from three different species of mushrooms was investigated in terms of starch characteristics (content, gelatinisation, and digestibility) and antioxidant capacities. The decrease in total starch contents, and increase in phenolic contents of the breads, were associated with increased mushroom powder contents. Mushroom inclusion reduced the rate of reducing sugar released over 120 min in an in vitro digestion compared to the control sample, implying a lower area under the curve (AUC) value with the inclusion of mushroom powder and a potentially lower predicted glycaemic response of the bread. Mushroom powder incorporation also enhanced the DPPH radical scavenging assay and oxygen radical absorbance capacity (ORAC) compared to control bread. The action of the addition of different mushroom powders on the bread crust and crumb microstructure properties was also studied. Mushroom powder altered the internal microstructure of the bread crust and crumb by affecting the interactions between starch and the other components of the bread. Overall, this shows that mushroom powder could be added to bread to deliver health benefits to consumers.

## 1. Introduction

There are a wide range of bakery products available to consumers, among which bread is regarded as an important dietary food in the majority of countries worldwide. The main ingredient used for bread making is wheat flour, which is mostly composed of starch (contributing 78–88% of the dry mass of the flour) [1]. Bread makes an important contribution to the carbohydrate intake in human diets. Commercial wheat bread is carbohydrate rich, which causes a quite rapid rise in blood glucose [2]. Recently, however, consumers have desired healthier food products, and this has meant that composite flours, partially substituted with other natural nutritional ingredients (including vitamins, minerals, dietary fibre and antioxidants), have attracted the interest of producers and consumers for distributing more health benefits.

Mushroom fruiting bodies have been used for food, in food flavouring materials and in traditional Chinese medicine for centuries. Mushrooms are a good source of high-quality digestible protein (10–40% dry mass), B vitamins, vitamin C and essential minerals [3,4]. Most mushroom species are rich in lysine, which is deficient in wheat flour [3]. Edible mushrooms have several medical properties, for instance, antitumor activity related to their β-D-glucan content [4,5]; antidiabetic from decreasing blood glucose related to their bioactive components; and anti-cardiovascular and anti-atherosclerotic effects due to their range of antioxidant components (including phenolics and ergothioneine) [6,7]. Thus, combinations of mushroom powder and wheat flour may possibly optimise bread formulations by improving their nutritional benefits, taste, flavour, and product appeal. Previous researchers have studied the effect of shiitake stipe, silver ear, mushroom mycelia, maitake mushroom and *Pleurotus eryngii* mushroom powder [5,8,9] on bread properties.

In this study, white button (*Agaricus bisporus*), shiitake (*Lentinula edodes*), and porcini mushrooms (*Boletus edulis*) were incorporated into wheat bread at 5%, 10% and 15% substitution levels. The influence of such inclusions on the nutritional characteristics and microstructure of breads were determined, and included total starch content, starch gelatinisation, in vitro starch digestibility, total phenolic contents, and antioxidant capacities. The microstructure properties of both the bread crust and crumb were evaluated by scanning electron microscopy. These final novel breads may introduce more edible mushroom species to Western consumers’ diets in an acceptable way through simple preparation.

## 2. Materials and Methods

### 2.1. Materials

High grade white flour (Champion, Auckland, New Zealand), white sugar (Chelsea, Auckland, New Zealand), iodised table salt (Cerebos, Auckland, New Zealand), yeast (Edmonds, Auckland, New Zealand) and butter (Pams, Auckland, New Zealand) were obtained from the local supermarket. Dried shiitake mushroom and porcini mushroom sliced (Jade Phoenix, Guangzhou, China) were used in this study together with fresh white button mushroom obtained from Meadow Mushrooms (Christchurch, New Zealand).

### 2.2. Bread Preparation

Mushroom powders were prepared according to the method reported by [10]. The bread samples were made according to the straight dough method (AACC, 2000). The recipe consisted of wheat flour (500 g), granulated sugar (30 g), salt (7.5 g), yeast (7.5 g), fat (25 g) and water (300 mL).

The dough was formed by using a mixer (Brabantia, Eindhoven, Netherlands) to combine wheat flour, mushroom powder, dry ingredients, yeast and water at speed 1 for 5 min. The speed of the mixer was increased to 3 for another 10 min until elastic dough was formed. The dough was rested for 30 min, then hand-kneaded for 5 min and left for 15 min. Dough pieces were divided (100 g), manually rounded, rolled and put into baking pans greased with margarine and placed in a proofer (Sanyo, Osaka, Japan) at 40 °C for 60 min. The bread was baked in an electric oven at 200 °C for 15 min. The baked bread loaves were allowed to cool for 2 h for subsequent analysis.

**Figure 1:**
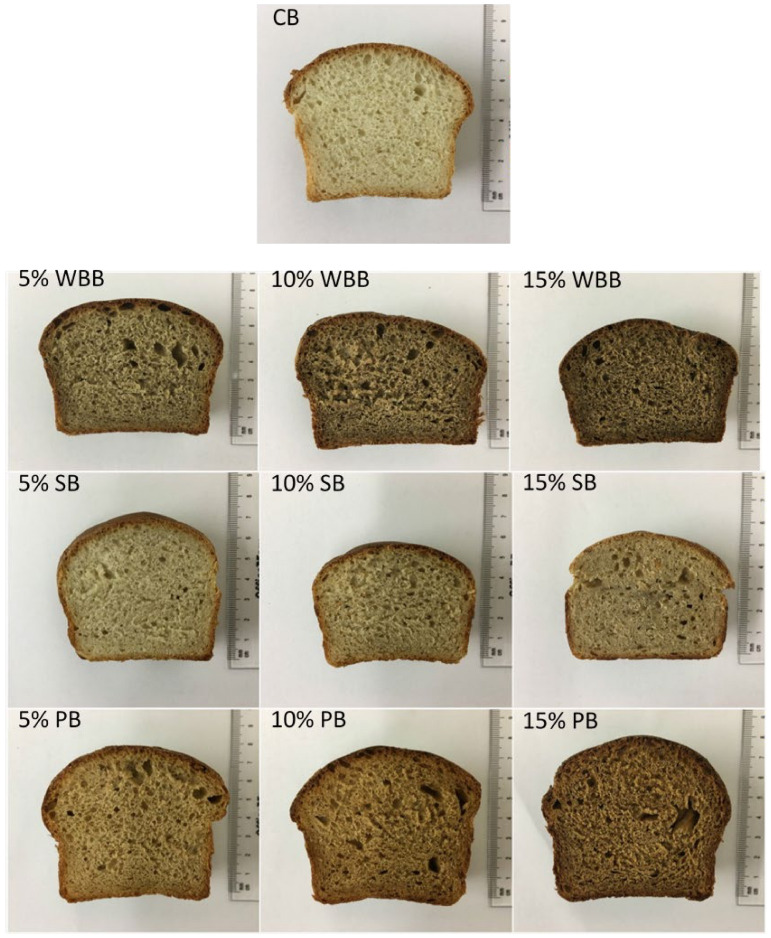
Slices of the control bread (CB) and all mushroom powder enriched bread samples: white button mushroom bread (WBB); shiitake mushroom bread (SB) and porcini mushroom bread (PB).

Nine different samples supplemented with mushroom powder were produced substituting four with white button mushroom, shiitake mushroom and porcini mushroom powder, respectively. Mushroom powder enriched formulations replaced wheat flour at levels of 5, 10, and 15 g/100 g (*w*/*w*), respectively. The control reference sample was prepared using wheat flour only (Figure 1).

### 2.3. Fibre Analysis

Total dietary fiber (TDF) content was determined in duplicate using a Total Dietary Fiber assay kit (Megazyme International Ireland Ltd., Wicklow, Ireland) and measurements were recorded for soluble (SDF) and insoluble fibre (IDF) composition as described by [11].

### 2.4. Thermal Properties

Differential scanning calorimetry (DSC; TA Q20, TA Instruments, Newcastle, DE) was employed to measure the starch gelatinisation characteristics of bread samples by measuring the temperature at which different stages of the process occur. Ground samples (4–6 mg) were weighed into a hermetic DSC pan and 10 μL of water was added. The sample was hermetically sealed and allowed to equilibrate overnight at room temperature. An empty pan and lid were used as reference. Duplicate thermal scans were performed from 10 to 110 °C at a rate of 10 °C/min. Temperatures of onset (Tonset), gelatinisation (peak), endset (Tendset) and the enthalpy of the transition (ΔH) were obtained in this process [12].

### 2.5. Starch Content and In Vitro Digestion Analysis

Total starch was determined according to AOAC Official Method 996.11, using Amyloglucosidase–α-Amylase Method. This method was conducted using the Megazyme Starch analysis kit (Megazyme International Ireland Ltd., Wicklow, Ireland).

The potential amount of glucose released over 120 min was conducted in triplicate using a multi-enzyme in vitro digestion method for each bread sample as described previously [13].

### 2.6. Antioxidant Analysis

Total phenolic content (TPC) of all samples was measured using 0.2 N Folin-Ciocalteu reagent (Sigma, St Louis, MI, USA) according to the method reported by [14]. The results were expressed as gallic equivalent per gram dry weight. The ability of all the samples to scavenge DPPH (1,1-diphenyl-2-picrylhydrazyl) radical was determined by the method adapted by [15]. The results were expressed as micromoles of Trolox per gram dry weight. The oxygen radical absorbance capacity-fluorescence assay was carried out using the method described by [16]. The results were expressed as micromoles of Trolox per gram dry weight.

### 2.7. Microstructure

The microstructure analysis of bread crust and crumb was evaluated by scanning electron microscope (SEM) on samples. The crust and crumb samples were freeze-dried prior to the analysis, then coated with gold using a MCI000 Ion Sputter Coater (Hitachi, Japan). Samples were viewed using a SU8010 SEM (Hitachi, Tokyo, Japan) at 1000× magnification level.

### 2.8. Statistical Analysis

Unless stated elsewhere, experiments were performed in triplicate. Statistical differences were determined by one-way analysis of variance (ANOVA) and Tukey’s comparison test (*p* < 0.05). Pearson’s correlations were also carried out to analyse the significant correlations at *p* ≤ 0.05, *p* ≤ 0.01, and *p* ≤ 0.001, respectively.

## 3. Results and Discussion

### 3.1. Effect of Mushroom Powder on the Thermal Properties of Bread

Previous research has shown that variations in gelatinisation temperatures within a dough and bread sample matrix may be important factors to predict bread quality [17]. The melting enthalpy (ΔH) was determined for all bread samples to establish the extent of the starch granule gelatinisation, or dextrinisation, during the bread making process [17]. Table 1 illustrates that the ΔH values of most of the mushroom bread samples were higher than those of the control, and these values increased with the increasing mushroom powder contents. Supplementary Materials Table S1 illustrates that the inclusion of mushroom powder significantly increased the fibre content of the bread. For instance, it has been reported that the limited levels of fibre and sugars in the breads made for greater water availability than for starch alone and this contributed to its complete gelatinisation and that this in turn may result in an increased ΔH [12]. Table 2 shows that there were significant positive correlations between ΔH and all IDF, SDF, and TDF (*r* = 0.580, 0.620 and 0.653, respectively, at *p* ≤ 0.001). Such a phenomenon may be due to the limited water content during bread making and competitive action by all the hydrophilic components in the mushroom powder being able to retard the starch granules’ swelling. A similar observation regarding the reduced degree of gelatinisation has been recorded [12]. As pectin and mushroom contain some specific lectins, such as agglutinin, it is possible that the presence of these hydrocolloids could reduce the hydration of starch [18].

### 3.2. The Effect of Mushroom Powder on the Starch Content and Digestibility of Bread

**Figure 2:**
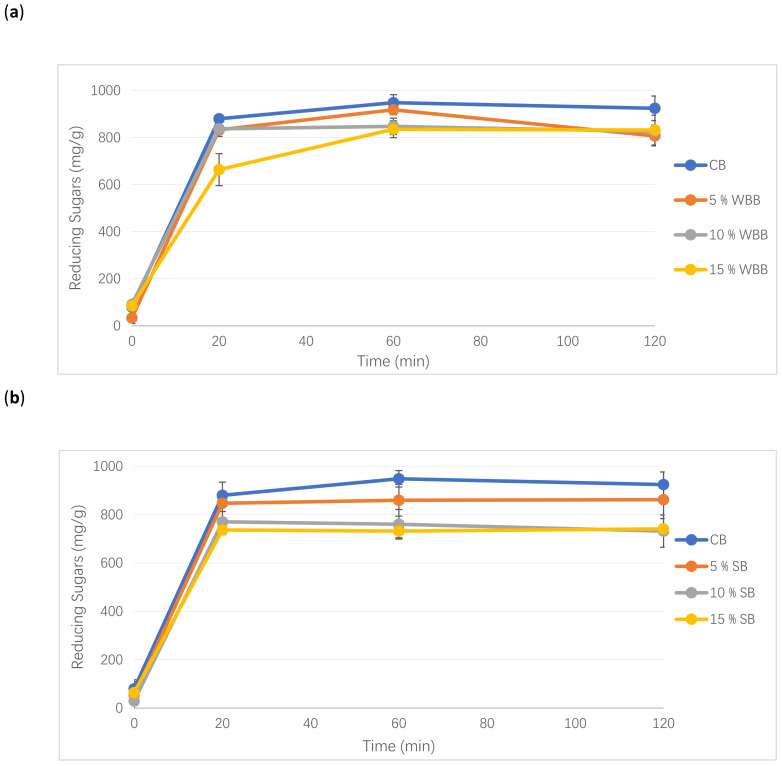

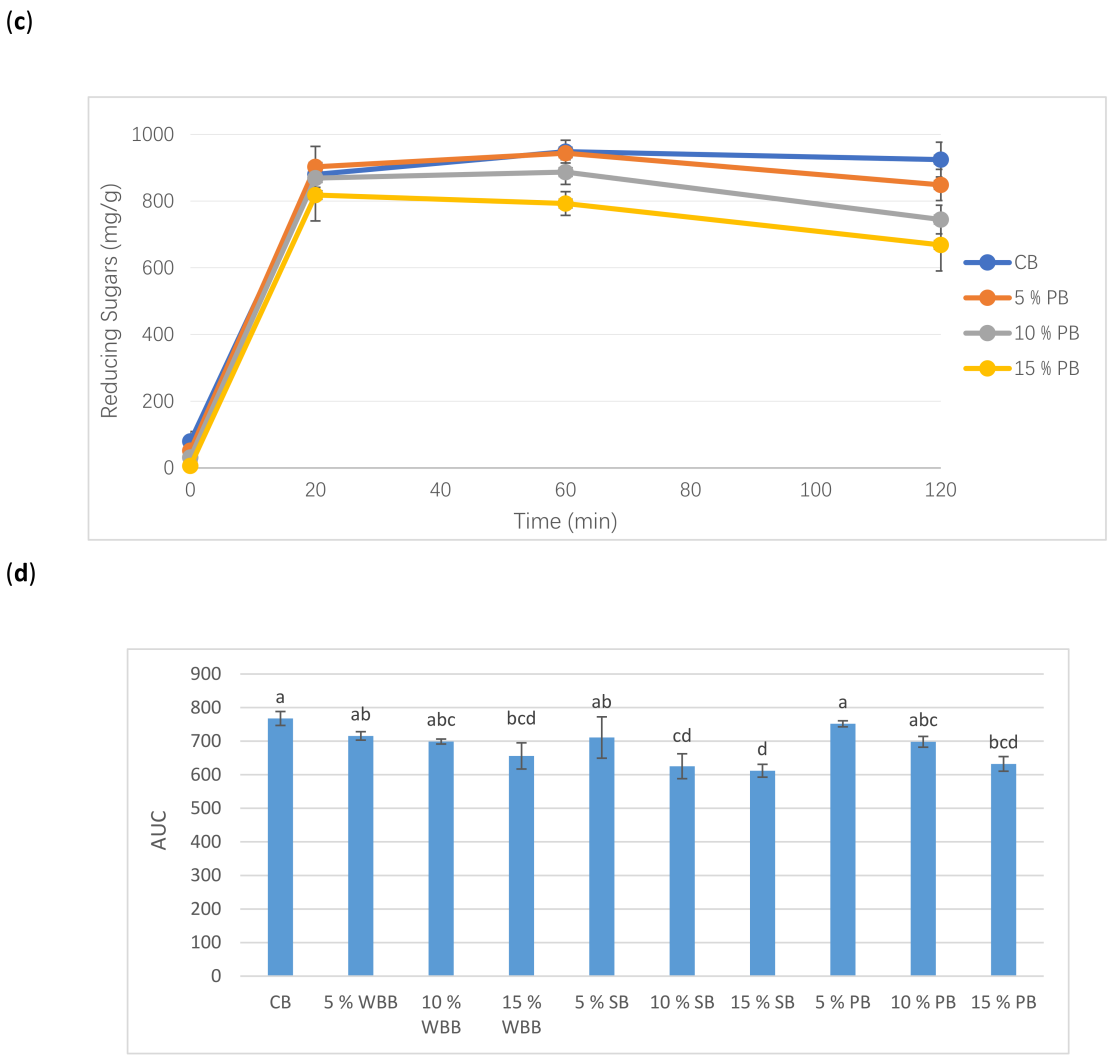
Levels of reducing sugars released during in vitro digestion. Comparing the control to 5%, 10%, 15% white button mushroom bread (**a**); shiitake mushroom bread (**b**); and porcini mushroom bread (**c**). Values for area under the curve (AUC) (**d**): white button mushroom bread (WBB); shiitake mushroom bread (SB) and porcini mushroom bread (PB). Error bars represent standard deviation of replicates. The same letter is not significantly different from each other (*p* < 0.05).

Supplementary Materials Figure S1 illustrates the total starch content of bread samples. The substitution of wheat flour with mushroom powder caused a decrease in the total starch content (dry basis). Negative correlations were observed between starch content and IDF (*r* = −0.618; *p* ≤ 0.001), SDF (*r* = −0.365; *p* ≤ 0.05), and TDF (*r* = −0.608; *p* ≤ 0.001) (Table 2). Figure 2 illustrates the reducing sugars released over a 120 min in vitro bread digestion. Lower levels of reducing sugars were released from all the mushroom enriched breads compared to the control. The levels of reducing sugars in the control bread were significantly higher, at 60 and 120 min, in the in vitro digestion compared to the mushroom enriched breads. The strongest decrease in reducing sugar release was observed in the 15% substitution level. The rapidly digested starch during the first 20 min in the mushroom enriched samples was reduced compared to the control. The amounts of the slowly digested starch fractions, over 20–120 min, were lower in the mushroom bread samples when compared to the control. Figure 2d represents the influence of mushroom powder inclusions on the standardized AUC bread values compared to the control sample. Increasing mushroom powder substitution levels decreased the AUC values. The values of the 15% mushroom bread samples and 10% shiitake mushroom bread were significantly lower than the control. These phenomena might be partly attributed to the significantly lower total starch content (Supplementary Materials Figure S1), as well as the reduced degree of starch gelatinisation (Table 1) and more resistant starch levels in the mushroom breads than in the control. A significant positive correlation existed between the total starch content and AUC value (*r* = 0.699, at *p* ≤ 0.001), whereas a negative correlation existed between ΔH and both the total starch content and AUC (*r* = −0.773 and −0.586, respectively, at *p* ≤ 0.001).

The nature of starch (granule size and amylose/amylopectin ratio) plays a principal role in the glycaemic response of breads [19], in that smaller starch granules with a high specific surface area are susceptible to enzymatic attack, thus increasing the rate of starch digestibility. Starch gelatinisation can lead to reduced crystallinity during bread baking, which makes the starch granules easier to digest by the endogenous enzymes [20]. Table 1 shows that the addition of mushroom powder inhibited starch gelatinisation of the bread. The composition of starch is also an important factor in the starch hydrolysis rate. It has been reported that amylose molecules form a compact amylose matrix by recrystallization, that then limits the mobility of the enzymes [19]. Compared to amylose, amylopectin molecules have a large surface area, which makes them more enzymatically susceptible [21]. Previous research has illustrated that fibre-enriched flour affects starch digestibility through fibre associating with amylose [19].

Dietary fibre is resistant to the digestion within the human small intestine and benefits the ‘good’ microfauna growth though fermentation in the human colon [1]. Several researchers have investigated the effect of the inclusion of fibre on the glycaemic response of foods [1,13,22]. The three species of mushroom powder used in this study have much higher total dietary fibre contents than wheat flour. Table 2 illustrates significant negative correlations between AUC and all IDF, SDF, and TDF (*p* ≤ 0.001, 0.05 and 0.001, respectively). Interestingly, compared with SDF, both IDF and TDF played more important roles in modulating the starch digestibility of the final bread samples. This may be attributed to the fact that fibre has the capacity to protect the starch granules from human physical digestive actions inside the stomach by increasing the viscosity of the digesta and form a physical “barrier” to the digestion of starch granules [1,22]. In addition, dietary fibre could form a close and compact matrix with other ingredients in bread, such as protein and starch, and such complex formations play a pivotal role in inhibiting enzyme accessibility to starch granules [22,23].

As determined in our previous work [10], the three mushroom powders used in this experiment contained more protein than wheat flour, which may be another reason for the reduced glycaemic response of the mushroom enriched breads. It has been observed that even small amounts of protein in food products were enough to alter the starch digestibility and other functional properties [21] in that the protein matrix is able to entrap the starch granules and so reduce accessibility to enzyme attack. Continuous protein strands have an encapsulation effect on large starch granules, resulting in well-formed protein-starch complexes; thus, limiting starch degradation and sugar liberation [23].

The results of our previous work show that the fat content of all three mushroom species powders were higher than those of wheat flour. It has also been shown that the proportion of polyunsaturated fatty acids in the mushroom fat can be as high as around 75%; including 68.8–84% linoleic acid; 19.2% palmitic acid and 8.3% oleic acid [24]. Ref [21] illustrated that the inclusion of palmitic and oleic acids was a way to limit amylose accessibility to hydrolysis enzymes by 35% inhibiting gelatinisation, delaying retrogradation and reducing susceptibility to digestive enzymes.

**Figure 3:**
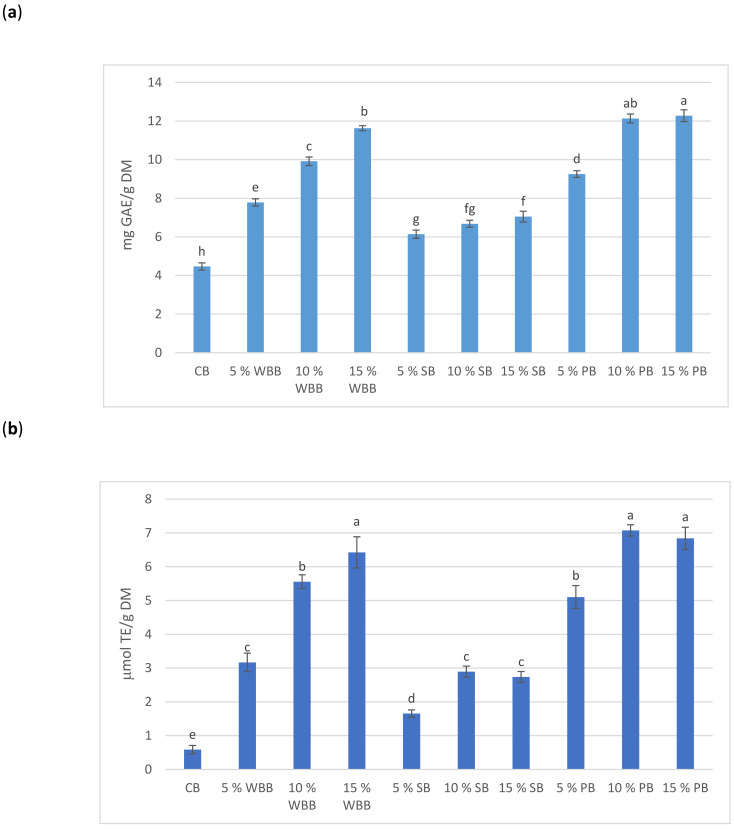

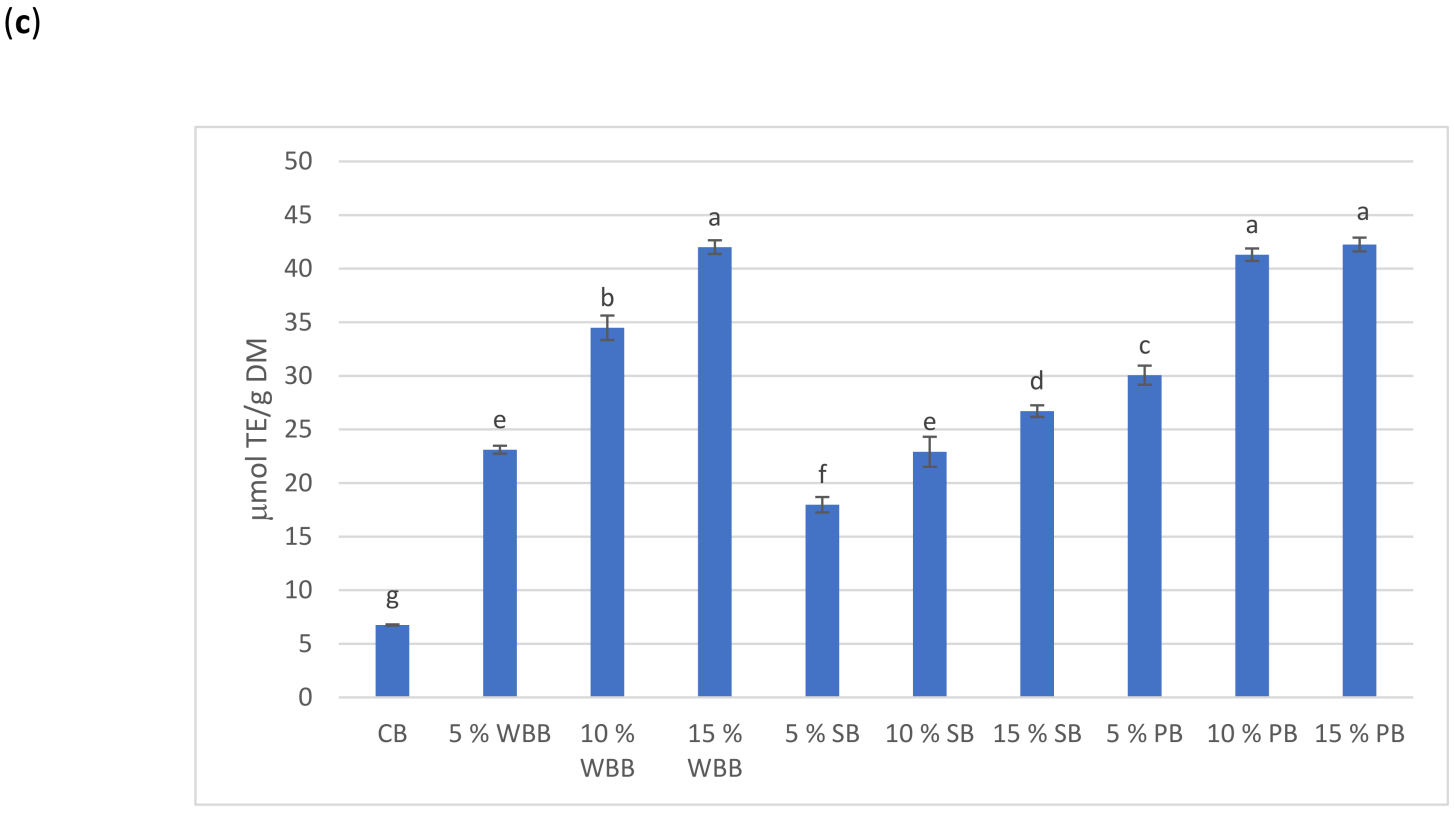
Values for total phenolic component (TPC) (**a**); antioxidant capacities: the DPPH. scavenging activities (**b**); and the ORAC assay results (**c**). Comparing the control bread (CB) to all mushroom powder enriched bread samples: white button mushroom bread (WBB); shiitake mushroom bread (SB) and porcini mushroom bread (PB). Error bars represent standard deviation of replicates. The same letter is not significantly different from each other (*p* < 0.05).

Oleic and linoleic acids in the mushroom extracts have been shown to have a strong anti-α-glucosidase capability [25]. Furthermore, edible mushrooms are a good source of organic acids and contain at least five organic acids, in turn organic acids limit starch digestibility and, subsequently, decrease glycaemic response of foods [26]. This effect may be attributed to the ability of organic acids to slow the gastric emptying rate and strengthen the protein-starch formation [20]. In addition, polyphenolic compounds or phenolic acids in the added mushroom powder (Figure 3a) could act as non-proteinaceous amylase inhibitors [21]. Ref [27] reported that polyphenols reduced starch hydrolysis by binding with amylases.

### 3.3. The Effect of Mushroom Powder on the Total Phenolic Contents and Antioxidant Properties of Bread

Figure 3 shows the total phenolic content, DPPH radical-scavenging and the oxygen radical absorbance capacity (ORAC) of the fortified breads. Bread made with 100% high grade wheat flour had the lowest total phenolic, DPPH radical-scavenging and ORAC levels of all samples. Mushroom powder substituted for wheat flour enhanced the total phenolic content significantly and the antioxidant capacities of the fortified bread samples. Table 2 shows significant positive correlations between TPC and both DPPH and ORAC (*r* = 0.960 and 0.979, respectively; at *p* ≤ 0.001), and between DPPH and ORAC (*r* = 0.966, *p* ≤ 0.001).

Bread production processing, including mixing, fermentation, and baking, has been shown to influence the final phenolic contents and activity with research indicating that the antioxidant capacity of bread decreased after baking [28]. This observation was mainly due to the baking high temperature that led to a decrease in, or oxidation of, some of the antioxidant compounds that were not thermally stable. Thermal treatment may also liberate part of the insoluble bound phenolics and enhance the antioxidant properties [29]. In addition, the products of the Maillard reaction are able to affect the antioxidant capacity of bread [28]. The total phenolic contents of bread were about twice as high as that of flour, possibly due to the positive ability of dough fermentation on the release of phenolics from flour. Mushrooms have always been regarded as a good source of phenolic compounds, which has been associated with their antioxidant, anti-inflammatory, or anti-tumour abilities. Mushrooms contain antioxidant compounds, including organic acids, alkaloids and ergothioneine [30]. In this study, the mushroom powders increased the level of bioactive components in the final breads, even after fermentation and baking, increased the antioxidant capacities of the final products.

Except for the white button mushroom breads, there were no significant differences between the 10 and 15% substitution levels for the DPPH and ORAC assay results in porcini mushroom breads and in the DPPH of shiitake breads (Figure 3), which may due to the same trend as their total phenolic contents results. Researchers have indicated that the occurrence of formation between phenolic and other components during the bread making process may lower the phenolic bioaccessibility index of the phenolic fortified bread [29]. Therefore, the interaction of mushroom phenolics with wheat components (such as starch and protein) acted as one of the most important factors in the antioxidant activity of the final breads, and can include the phenolic-phenolic, phenolic-starch and phenolic-protein matrices. Ref [31] indicated that the formation between phenolics (extracted from tea, coffee and cocoa) and milk proteins favoured the reduction in the free phenolic contents and negatively correlated with the antioxidant activity of dairy products. Table 2 demonstrates the strong positive correlations between TPC and all IDF, SDF and TDF (at *p* ≤ 0.05, 0.001, and 0.001, respectively), between DPPH and both SDF and TDF (*p* ≤ 0.001 and 0.01, respectively), and between ORAC and all IDF, SDF and TDF (at *p* ≤ 0.01, 0.001, and 0.001, respectively). This indicates a synergistic effect of dietary fiber and antioxidant activity possibly through carrier bioactive compounds.

It is of added interest is that there were significant and positive correlations between ΔH and all TPC, DPPH and ORAC (*r* = 0.493, 0.484 and 0.551, respectively, at *p* ≤ 0.01), whereas negative correlations existed between AUC and ORAC (*r* = −0.412; *p* ≤ 0.05), the starch content and all TPC, DPPH and ORAC (*r* = −0.409, −0.380 and −0.490, respectively; at *p* ≤ 0.05, 0.05 and 0.01, respectively). The results of the in vitro starch digestibility and the antioxidant properties of mushroom enriched breads are in agreement with the work of [29], who added quinoa leaves (a bioavailable phenolic-rich material) to bread and found that the phenolic-protein matrix might lead to blocking of the substrate and/or limitation enzymes. Similarly, green leafy vegetables are good sources of antioxidants and can lower starch digestion by phenolic-starch interaction.

### 3.4. The Effect of Mushroom Powder on the Microstructure of the Bread Crust and Crumb

**Figure 4:**
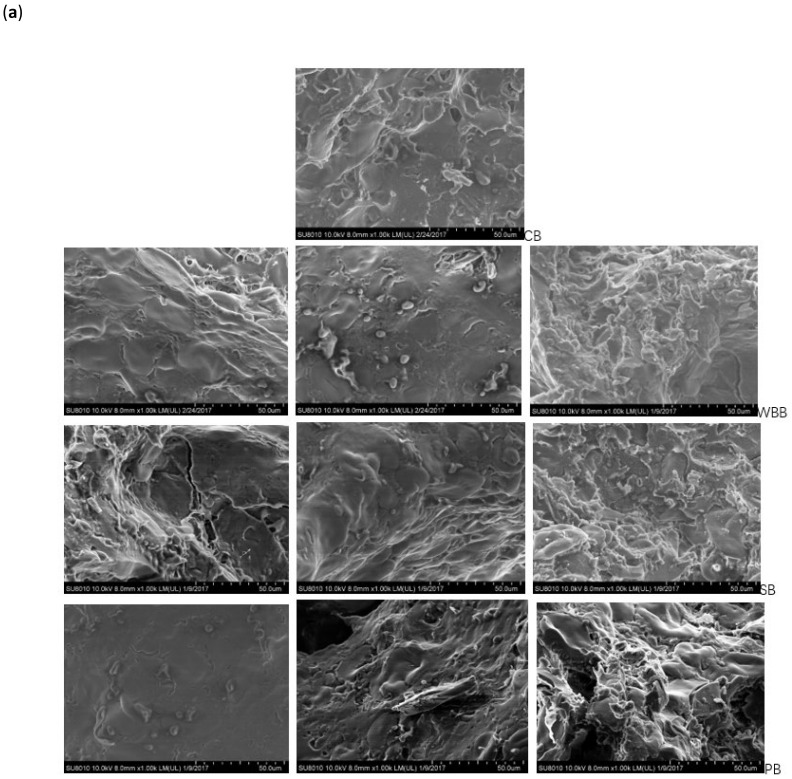

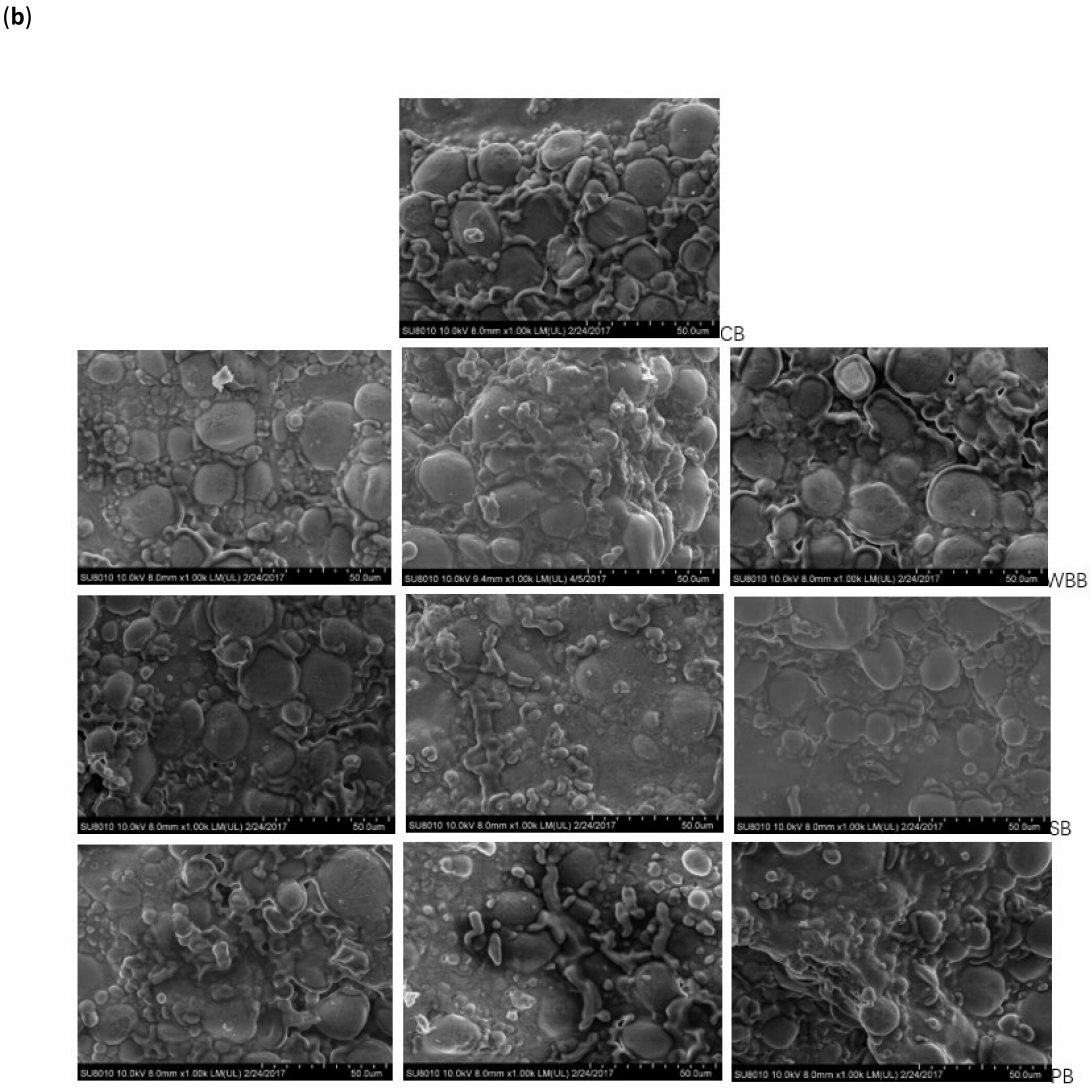
Scanning electron micrographs of bread crumb (**a**) and bread crust (**b**) at 1000× magnification: the control bread (CB); white button mushroom bread (WBB); shiitake mushroom bread (SB) and porcini mushroom bread (PB). From left to right: 5%; 10% and 15% substituent level.

Scanning electron microscopy of the control and all mushroom enriched bread crumbs and crusts are shown in Figure 4a,b, respectively. All these pictures illustrate the effect of the inclusion of mushroom powder on the morphological changes in bread structure during the whole bread making process. The small starch granules were observed as spherical or oval shapes that protruded from the gluten matrix and the large granules, lenticular in shape, were dispersed in the gluten-protein matrix [32]. Figure 4a shows that the starch-protein matrix of the control bread crumb was well formed and there was a strong and complex connection in all the components, with some small cavities. With the increase in mushroom supplementation, some large starch granules were seen to move to the exterior surface, which might reflect their lower degree of gelatinisation, and this observation was in agreement with the results of DSC (Table 1) and is similar to the observations regarding the surface starchy granules observed in protein matrix recorded by [33]. These factors may be related to the limited starch digestibility of the mushroom fortified breads. Increasing amounts of both white button and porcini mushroom made the crumb structures more porous, rough and less dense, while for the shiitake mushroom additions, the crumb structure started to be less compact from the 5% addition level upwards. All three mushroom species had higher insoluble dietary fibre, protein, and fat contents than wheat flour alone. It has been shown that insoluble dietary fibre could disrupt the gluten network and result in a discontinuous structure in the bread crumb [2]. In addition, the different levels and sizes of insoluble fibre from the different mushroom species could be considered as a reason for their different structures at the same substitution levels [2]. These observations about shiitake mushroom enriched breads may have contributed to their slightly higher insoluble fibre and lower protein amounts than the other two species. The 5% and 10% white button mushroom breads and the 5% porcini mushroom bread had a smooth surface and more compact appearance not dissimilar to the control (5% porcini mushroom bread looked even more compact than the control). This illustrated the continuous structure of the starch granules enveloped into the protein matrix. This might be due to the abundance of proteins the white button and porcini mushrooms provided to breads, which was supported by the findings of [23] who showed that protein could help develop a diffused and coagulated protein network to entrap starch granules. Compared with the control, the three mushroom supplemented samples showed a continuous sheet formation instead of wrapping, which may be attributed to the presence of large areas of sheet-like protein structures covering the starch granules [32]. In the case of breads with additional lipids, the structures tended to be smoother and nonporous without any phase separation [34]. Therefore, the antagonistic effects of additional insoluble fibre, protein and fat, and their interactions with starch granules could be responsible for the final different structures of the bread crumb.

Figure 4b shows the scanning electron micrographs of bread crust sections. The control crust layer represented a continuous veil-like film, with large starch granules and some small ones, which were slightly deformed, encapsulated completely and uniformly distributed in the lipoprotein network. In terms of the mushroom-enriched samples, loose starch granules were visible on the surface of the network with large areas of heterogeneous starch structures in a discontinuous phase. The starch granules possibly could, therefore, have altered the bread architecture in different ways [34]. The inclusion of mushroom powder made some starch granules lose their identity and aggregate in polymeric areas of protein.

## 4. Conclusions

This manuscript comprehensively illustrates that some of the nutritional quality attributes of conventional breads may be enhanced by the enrichment of bioactive ingredients from mushroom material. In particular, the research identifies significant correlations between mushroom bioactive addition and increased fibre, antioxidant, and phenolic contents, as well as correlations between fibre and phenolic composition of breads on the starch digestibility, starch gelatinisation, and antioxidant properties of breads. The 15% white button, 10% and 15% shiitake and 15% porcini mushroom enriched breads resulted in lower predictive glycaemic indexes than the control. Increased mushroom powder inclusion levels led to a progressive increase in the total phenolic content and antioxidant capacities for most samples (the 10% and 15% levels did not show any obvious difference). Furthermore, the mushroom powder disrupted the initial intact starch–protein matrix and reconstructed the bread crust and crumb microstructures. The SEM images indicate multiple interactions (synergistic or antagonistic) between the additional nutrients that the mushroom powder provided and the inherent constituents. It should be noted that in this research, we did not investigate the separate effects of bound and free phenolic compounds on the nutritional quality of the breads. Other researchers have endeavoured to determine the different effects of bound phenolic compounds and free phenolic compounds on starch and protein digestion, based on the understanding that the solubility and binding strengths of phenolic compounds affects the physical and chemical characteristics of fortified foods [35]. For instance, researchers have illustrated that free phenolic compounds contributed between 50–83% of total phenolic compounds in quinoa-based foods, indicating the importance of understanding the nature of free phenolic compounds in relation to antioxidant activity [36]. Researchers have illustrated that processing has a significant effect on the ratio of free and bound phenolic compounds present in foods based on water mobility and overall solubility of the system [37], and that the understanding of the relative composition of free and bound phenolic compounds in fruits (for instance) is of great importance with the overall contribution of antioxidant capacity of a material [38]. For this reason, it would be prudent for future research to investigate the relationship of free and bound mushroom phenolic compounds on the rate and extent of starch degradation. This way, it would be possible to determine any inhibitory and nutritional benefits that could be derived from either of the phenolic fractions separately, or whether the combination of both free and bound phenolic compounds yields a greater benefit to the consumer.

## Figures and Tables

**Table 1:** The effect of different mushroom powder on the thermal properties of bread.

-	Tonset °C	Tgelatinization °C	Tendset °C	ΔH J/g	ΔTr °C
Control bread	58.06 ± 0.84	64.41 ± 1.73	69.85 ± 1.17	0.42 ± 0.08	11.80 ± 0.33
5% White button mushroom bread	55.96 ± 1.07	60.14 ± 0.85	67.19 ± 2.51	0.38 ± 0.07	11.23 ± 3.59
10% White button mushroom bread	55.64 ± 2.17	59.62 ± 0.09	65.92 ± 2.19	0.40 ± 0.14	10.29 ± 0.02
15% White button mushroom bread	52.71 ± 1.70	59.84 ± 0.36	70.59 ± 0.68	0.86 ± 0.10	17.89 ± 2.38
5% Shiitake mushroom bread	56.34 ± 2.82	61.31 ± 0.28	68.79 ± 1.83	0.44 ± 0.08	12.45 ± 4.65
10% Shiitake mushroom bread	53.79 ± 0.81	61.16 ± 0.49	73.14 ± 0.60	0.73 ± 0.10	19.35 ± 0.21
15% Shiitake mushroom bread	52.97 ± 1.94	59.59 ± 1.58	68.31 ± 1.69	0.64 ± 0.09	15.34 ± 0.25
5% Porcini mushroom bread	54.2 ± 0.16	61.01 ± 0.07	69.88 ± 0.82	0.42 ± 0.02	15.68 ± 0.98
10% Porcini mushroom bread	54.30 ± 1.01	60.14 ± 0.80	68.93 ± 0.26	0.56 ± 0.03	14.63 ± 1.27
15% Porcini mushroom bread	52.74 ± 1.41	57.10 ± 0.73	67.13 ± 0.96	1.27 ± 0.2	14.39 ± 2.38

Mean ± standard deviation.

**Table 2:** Pearson’s correlation coefficient (*r*) of physicochemical and nutritional properties of bread samples.

-	ST	AUC	TPC	DPPH	ORAC	ΔH	IDF	SDF	TDF
ST	-	0.699 ***	−0.409 *	−0.380 *	−0.490 **	−0.773 ***	−0.618 ***	−0.365 *	−0.608 ***
AUC	-	-	−0.355	−0.232	−0.412 *	−0.586 ***	−0.858 ***	−0.368 *	−0.802 ***
TPC	-	-	-	0.960 ***	0.979 ***	0.493 **	0.432 *	0.752 ***	0.573 ***
DPPH	-	-	-	-	0.966 ***	0.484 **	0.342	0.764 ***	0.504 **
ORAC	-	-	-	-	-	0.551 **	0.509 **	0.776 ***	0.642 ***
ΔH	-	-	-	-	-	-	0.580 ***	0.620 ***	0.653 ***
IDF	-	-	-	-	-	-	-	0.539 **	0.968 ***
SDF	-	-	-	-	-	-	-	-	0.734 ***

ST, starch; AUC, in vitro area under the curve value; TPC, total phenolic content; DPPH, the DPPH. scavenging activities; ORAC, the ORAC assay results; ΔH, the enthalpy of the transition; IDF, insoluble dietary fibre; SDF, soluble dietary fibre; TDF, total dietary fibre. *, significant at *p* ≤ 0.05. **, significant at *p* ≤ 0.01. ***, significant at *p* ≤ 0.001.

## Data Availability

No applicable.

## Supplementary Materials

The following are available online at https://www.mdpi.com/article/10.3390/foods10040731/s1, Figure S1: The total starch content, Table S1: Fibre content of bread products.
