# Supplementary material for: Enhancing the Nutritional Properties of Bread by Incorporating Mushroom Bioactive Compounds: The Manipulation of the Pre-Dictive Glycaemic Response and the Phenolic Properties
Source: Foods. 2021 Mar 30;10(4):731. doi: 10.3390/foods10040731 (PMC8066703; doi:10.3390/foods10040731)
Supplement: Supplementary file 1 [file foods-10-00731-s001.pdf]

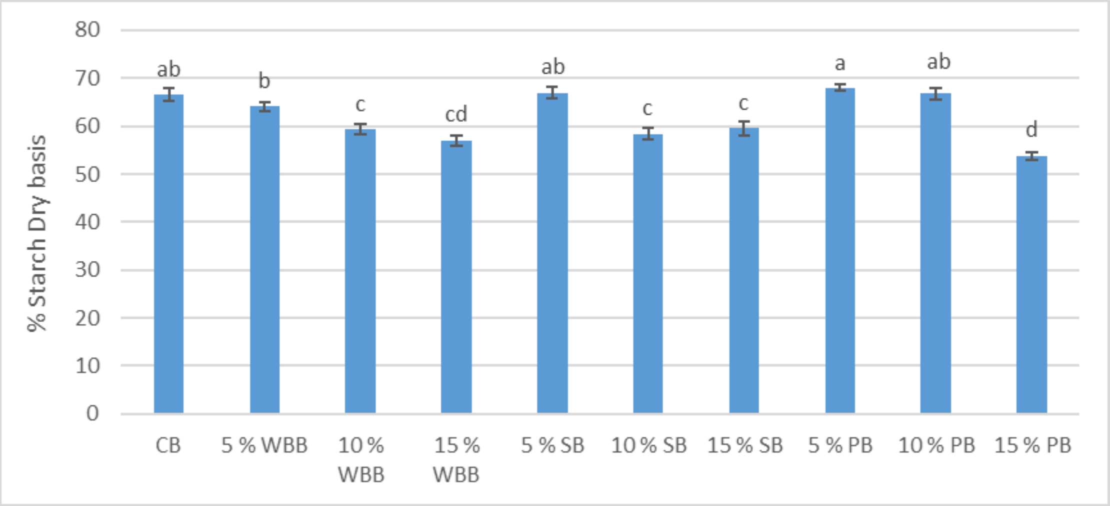

Figure S1. The total starch content. Comparing the control to 5 %, 10 %, 15 % white button mushroom bread (**a**); shiitake mushroom bread (**b**); and porcini mushroom bread (c): white button mushroom bread (WBB); shiitake mushroom bread (SB) and porcini mushroom bread (PB). Error bars represent standard deviation of replicates. The same letter is not significantly different from each other ($p < 0.05$).

Table S1. Fibre content of bread products.

-	IDF %	SDF %	TDF %
Control bread	2.24±0.17D	2.67±0.085B	4.91±0.087D
5 % White button mushroom bread	2.55±0.33D	2.81±0.23AB	5.36±0.10CD
10 % White button mushroom bread	3.50±0.28BCD	3.12±0.20AB	6.62±0.48BC
15 % White button mushroom bread	4.02±0.82ABC	3.20±0.060AB	7.23±0.76AB
5 % Shiitake mushroom bread	3.29±0.024BCD	2.88±0.54AB	6.16±0.52BCD
10 % Shiitake mushroom bread	4.44±0.18AB	3.09±0.24AB	7.53±0.062AB
15 % Shiitake mushroom bread	5.19±0.30A	3.28±0.020AB	8.47±0.28A
5 % Porcini mushroom bread	2.88±0.07CD	3.36±0.19AB	6.24±0.12BCD
10 % Porcini mushroom bread	3.99±0.47ABC	3.57±0.14A	7.56±0.34AB
15 % Porcini mushroom bread	4.39±0.0071AB	3.69±0.0064A	8.07±0.014A

Mean ± standard deviation. Values within a vertical column followed by the same letter are not significantly different from each other ($p < 0.05$).
